# Inhaled 45–50% argon augments hypothermic brain protection in a piglet model of perinatal asphyxia

**DOI:** 10.1016/j.nbd.2015.12.001

**Published:** 2016-03

**Authors:** Kevin D. Broad, Igor Fierens, Bobbi Fleiss, Eridan Rocha-Ferreira, Mojgan Ezzati, Jane Hassell, Daniel Alonso-Alconada, Alan Bainbridge, Go Kawano, Daqing Ma, Ilias Tachtsidis, Pierre Gressens, Xavier Golay, Robert D. Sanders, Nicola J. Robertson

**Affiliations:** a Institute for Women's Health, University College London, United Kingdom; b Centre for the Developing Brain, Kings College, St Thomas's Campus, London, United Kingdom; c Inserm, Paris, France; d University Paris Diderot, Sorbonne Paris Cite, UMRS, 1141 Paris, France; e Physics and Bioengineering, University College London NHS Trust, London, United Kingdom; f Department of Anaesthetics, Intensive Care and Pain Medicine, Department of Surgery & Cancer, Imperial College London, Chelsea and Westminster Hospital, London, United Kingdom; g Department of Medical Physics and Biomedical Engineering, University College London, United Kingdom; h Institute of Neurology, University College London, United Kingdom; i Department of Anesthesiology, University of Wisconsin, Madison, United States

**Keywords:** Argon, Neonatal encephalopathy, Therapeutic hypothermia, Noble gas, Hypoxia–ischemia

## Abstract

Cooling to 33.5 °C in babies with neonatal encephalopathy significantly reduces death and disability, however additional therapies are needed to maximize brain protection. Following hypoxia–ischemia we assessed whether inhaled 45–50% Argon from 2–26 h augmented hypothermia neuroprotection in a neonatal piglet model, using MRS and aEEG, which predict outcome in babies with neonatal encephalopathy, and immunohistochemistry. Following cerebral hypoxia–ischemia, 20 Newborn male Large White piglets < 40 h were randomized to: (i) Cooling (33 °C) from 2–26 h (n = 10); or (ii) Cooling and inhaled 45–50% Argon (Cooling + Argon) from 2–26 h (n = 8). Whole-brain phosphorus-31 and regional proton MRS were acquired at baseline, 24 and 48 h after hypoxia–ischemia. EEG was monitored. At 48 h after hypoxia–ischemia, cell death (TUNEL) was evaluated over 7 brain regions. There were no differences in body weight, duration of hypoxia–ischemia or insult severity; throughout the study there were no differences in heart rate, arterial blood pressure, blood biochemistry and inotrope support. Two piglets in the Cooling + Argon group were excluded. Comparing Cooling + Argon with Cooling there was preservation of whole-brain MRS ATP and PCr/Pi at 48 h after hypoxia–ischemia (p < 0.001 for both) and lower ^1^H MRS lactate/N acetyl aspartate in white (p = 0.03 and 0.04) but not gray matter at 24 and 48 h. EEG background recovery was faster (p < 0.01) with Cooling + Argon. An overall difference between average cell-death of Cooling versus Cooling + Argon was observed (p < 0.01); estimated cells per mm^2^ were 23.9 points lower (95% C.I. 7.3–40.5) for the Cooling + Argon versus Cooling. Inhaled 45–50% Argon from 2–26 h augmented hypothermic protection at 48 h after hypoxia–ischemia shown by improved brain energy metabolism on MRS, faster EEG recovery and reduced cell death on TUNEL. Argon may provide a cheap and practical therapy to augment cooling for neonatal encephalopathy.

## Highlights

• 45–50% Argon augments brain protection above hypothermia after neonatal hypoxia–ischemia.
• No physiological or biochemical change occurred with the 24 h exposure of 45–50% Argon.
• 45–50% Argon led to faster recovery of aEEG, and improved cerebral metabolism on MRS.
• TUNEL + cells were ~ 24 points lower per mm^2^ with Argon augmented cooling v cooling.
• Argon was practical to administer with a standard neonatal ventilator (unlike xenon).

## Graphical abstract

**Figure:**
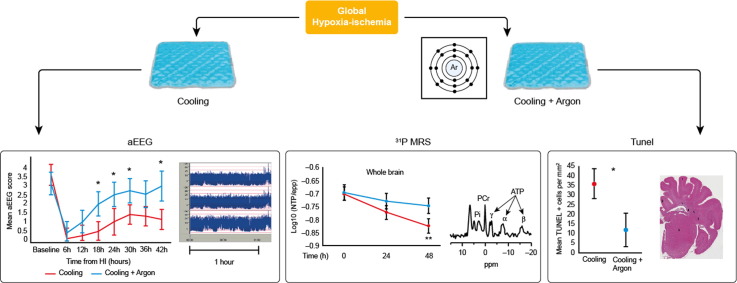

## 1 Introduction

Neonatal Encephalopathy (NE) consequent on perinatal hypoxia–ischemia is the third leading cause of child death and one of the main causes of preventable child neurodisability worldwide (Lawn et al., 2014). In the developed world, cooling to 33–34 °C for 72 h in moderate to severe NE increases the rate of survival without impairments in childhood to 15%, but despite cooling, around 25% infants die and 20% survivors have sensorimotor or cognitive impairments (Azzopardi et al., 2014). Attempts to increase brain protection with deeper and longer cooling (Alonso-Alconada et al., 2015, Shankaran et al., 2014) suggest that current clinical cooling protocols are optimal and that other therapies that can augment hypothermic neuroprotection in NE are needed (Robertson et al., 2012).

In a comparative review of potential neuroprotective agents, the noble gas Xenon was rated in the top six, however there was concern over its cost and the requirement for specialized equipment for delivery and scavenging (Robertson et al., 2012). Xenon has shown neuroprotective properties in adult and neonatal (Chakkarapani et al., 2010, Faulkner et al., 2011, Ma et al., 2005) models of hypoxia–ischemia; this neuroprotection is stronger in neonatal models when Xenon is combined with cooling. In neonatal rat (Ma et al., 2005) and piglet (Chakkarapani et al., 2010, Faulkner et al., 2011) studies, the combination of Xenon with cooling provided neuroprotection while neither intervention alone was as effective. Current interest is turning towards Argon, which is the most abundant inert gas already widely used in industries and available at a cost 200 times lower than Xenon. Argon does not produce demonstrable anesthetic effects at atmospheric pressure and provides potent neuroprotection, at least equivalent to Xenon, in animal models of hypoxic–ischemic brain injury and *in vitro* using murine organotypic hippocampal slice cultures (Loetscher et al., 2009) and neuronal cultures (Jawad et al., 2009). In some studies Argon is superior to Xenon for organ protection from ischemia-reperfusion injury (Irani et al., 2011). *In vitro* models of cerebral ischemia and traumatic brain injury suggest that the optimum concentration of Argon for protection is 50% and the therapeutic window lasts up to 3 h (Loetscher et al., 2009). Argon 50% administered one hour after transient middle cerebral artery occlusion (MCAO) in adult rats provided significantly reduced infarct volumes and composite adverse outcomes (Ryang et al., 2011). Protection has also been observed in neonatal rodent models where 70% argon at 2 h after hypoxia–ischemia improved cell survival to naive levels and reduced infarct volume (Zhuang et al., 2012).

We hypothesized that Argon-augmented cooling would lead to better brain protection than cooling alone after a hypoxic–ischemic insult. Our aim was to assess whether 24 h of 45–50% Argon started 2 h after hypoxia–ischemia augments hypothermic neuroprotection in a piglet perinatal asphyxia model. This model replicates neonatal intensive care with meticulous monitoring and control of physiological and metabolic parameters. This model also has strong similarities to newborn infants with NE in terms of the timing of the evolution of injury after hypoxia–ischemia (Azzopardi et al., 1989, Lorek et al., 1994), pattern of injury, neuropathology and cerebral magnetic resonance spectroscopy (MRS) (Thayyil et al., 2010). The efficacy of Argon protection was assessed using: (i) Cerebral MRS biomarkers, proton (^1^H) MRS lactate/N acetyl aspartate (NAA) (Thayyil et al., 2010) and phosphorus-31 (^31^P) MRS for phosphocreatine/inorganic phosphate (PCr/Pi) and NTP/exchangeable phosphate pool (epp) (Azzopardi et al., 1989); (ii) aEEG background activity recovery over 48 h, a strong predictor of outcome in babies with NE (van Rooij et al., 2005); and (iii) Histological assessment of cell death using TUNEL at 48 h after hypoxia–ischemia.

## 2 Materials and methods

### 2.1 Sample size calculation

Our primary outcomes were cerebral lactate/NAA and NTP/epp. Previous work with our model suggested that the change in lactate/NAA during 48 h varied between normo- and hypothermic groups by 1.0 U, with a standard deviation of 0.65 U (log scale). Assuming similar magnitude of additional effect for Argon-augmented cooling following HI versus cooling alone and similar variability at 48 h and with 5% significance and 80% power, at least eight subjects were required in each group based on a two-sample t-test sample size calculation.

### 2.2 Animal experiments and surgical preparation

**Fig. 1:**
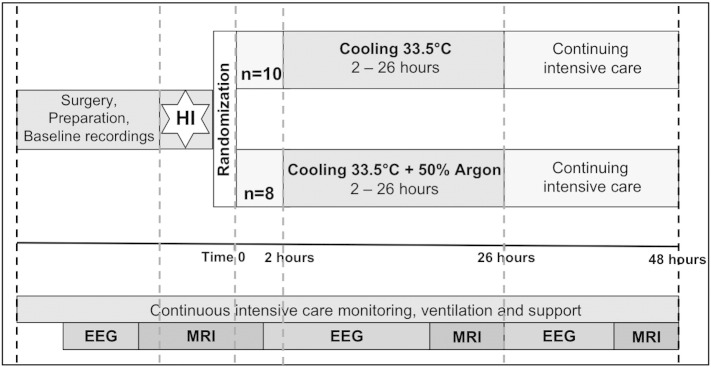
Study time-line. Following baseline data acquisition, piglets underwent cerebral hypoxia–ischemia. At the end of hypoxia–ischemia (time 0), piglets were randomized to (i) Cooling (33.5 °C) for 24 h or (ii) Cooling + 50% Argon for 24 h. Treatment was started at 2 h after Time 0. Piglets were maintained under meticulous intensive care for 48 h following HI, prior to euthanasia. MRS was acquired at baseline, during HI, for the first 60 min after HI, at 24 and 48 h. EEG was acquired at baseline and in between the MRS acquisitions.

All animal experiments were approved by the Ethics Committee of UCL and performed according to the UK Home Office Guidelines [Animals (Scientific procedures) Act, 1986]. The study complies with the ARRIVE guidelines. Twenty male piglets, aged less than 40 h, with weights 1.8–2.1 kg were anesthetized and surgically prepared as described previously (Lorek et al., 1994). The study time-line is shown in Fig. 1. Anesthesia was induced by 4% *v*/*v* isoflurane through a facemask for around 5 min to facilitate tracheostomy and intubation. Throughout the surgery, isoflurane was maintained at 2.8–3% guided by peripheral oxygen saturation monitoring (Nonin Medical, Plymouth, MN, USA) and the animal's response to stimulation. Following tracheostomy, a suitable size of endotracheal tube (Smiths Medical, Ashford, Kent, UK) was fixed and the piglet was mechanically ventilated (SLE 2000 infant ventilator, Surrey, UK). Ventilator settings were adjusted to maintain partial pressure of oxygen (PaO_2_) at 8–13 kPa and carbon dioxide (PaCO_2_) at 4.5–6.5 kPa, allowing for temperature and fraction of inspired oxygen (FiO_2_) correction of the arterial blood sample.

After the airway was secured, both common carotid arteries were surgically isolated at the level of the fourth cervical vertebra and a vascular occluder (OC2A, In Vivo Metric, Healdsburg, CA, USA) was placed on each side. After completion of surgery, inspired isoflurane concentration was maintained at 2% v/v.

An umbilical venous catheter was inserted for infusion of maintenance fluids (10% dextrose, 60 ml/kg/day before the insult and 40 ml/kg/day after resuscitation), fentanyl (5 μg/kg/h), and antibiotics (benzyl penicillin 50 mg/kg, every 12 h and gentamicin 4 mg/kg, once a day). An umbilical arterial catheter was inserted for invasive physiologic monitoring (SA instruments) for heart rate and arterial blood pressure, and blood sampling for arterial gases and electrolytes (Abbot Laboratories, UK). Hepsal (0.5 IU/ml of heparin in 0.9% saline solution) was infused at rate of 0.3 ml/h to prevent umbilical arterial catheter blockage.

Piglets were cared for under intensive care conditions throughout the experiment. To maintain the MABP above 40 mm Hg, bolus infusions of 0.9% saline (Baxter; 10 ml/kg), dopamine (5–20 μg/kg/min), dobutamine (5–20 μg/kg/min) and adrenaline (0.1–1.5 μg/kg/min) were used as required by a NICU trained clinician. High serum lactate was treated by optimizing oxygenation and 0.45% saline bolus infusions. Hyperkalemia (K > 7.0 mmol/l) was treated with 4 μg/kg salbutamol (10 μg/ml) over 10 min.

### 2.3 MR methods

The head was immobilized in a stereotactic frame for MRS acquisition. Piglets were positioned within the bore of 9.4 Tesla Agilent MR scanner. ^1^H and ^31^P MRS data were acquired at baseline and at 24 and 48 h after cerebral hypoxic-ischemia.

#### 2.3.1 ^31^P MRS

A 7 cm × 5 cm elliptical transmit-receive MRS surface coil tuned to the ^31^P resonant frequency was positioned on top of the head. ^31^P MRS was acquired with 1-minute resolution using a non-localized single-pulse acquisition. MRS data were analyzed using the Advanced Method for Accurate, Robust and Efficient Spectral fitting of MRS data with use of prior knowledge (AMARES) (Vanhamme et al., 1997) as implemented in the jMRUI software. Prior knowledge of NTP multiplet structure was used. NTP is predominately ATP and the latter contributes approximately 70% of the NTP signal (Mandel and Edel-Harth, 1966). Thus NTP changes during this experiment predominately reflected ATP changes. Pi was fitted using 4 separate components and PCr with a single component. The following peak-area ratios were calculated: Pi/epp, PCr/epp, and NTP/epp where epp = exchangeable phosphate pool = Pi + PCr + 2γ-NTP + β-NTP.

#### 2.3.2 ^1^H MRS

^1^H MRS data were collected from voxels located in the dorsal right subcortical white matter at the centrum semiovale level (white matter voxel, 8 × 8 × 15 mm) and in the deep gray matter centered on both lateral thalami (deep gray matter voxel, 15 × 15 × 10 mm) using a combination of a 65 × 55 mm elliptical receive surface coil, a 150 mm diameter transmit volume coil and a LASER acquisition (TR = 5000 ms, TE = 288 ms, 128 averages). Spectra were analyzed using AMARES as implemented in the jMRUI software and the lactate/NAA peak area ratio was calculated.

### 2.4 Cerebral hypoxia–ischemia (HI)

^31^P MRS data were collected at baseline, during hypoxia–ischemia and for one hour after cessation of hypoxia–ischemia. Hypoxia–ischemia was induced inside the MR scanner by remotely inflating the vascular occluders around both common-carotid arteries, and simultaneously reducing FiO_2_ to 6% (vol/vol). During hypoxia–ischemia the β-NTP peak height was continuously monitored using in-house Matlab (Mathworks) software. At the point at which β-NTP had fallen to 50% of its baseline value, FiO_2_ was increased to 9%. When β-NTP fell to 40% baseline height the inspired oxygen fraction was titrated to keep the β-NTP peak height between 30% and 40% of its original height for a period of 12.5 min. At the end of hypoxia–ischemia the carotid arteries were de-occluded and the FiO_2_ returned to 21%. Insult severity was calculated (Faulkner et al., 2011).

### 2.5 Experimental groups

Following resuscitation, while in the bore of the MR system, piglets were randomized (computer generated randomization revealed after HI) into 2 groups — Cooling or Cooling + Argon (Fig. 1). Both groups were cared for over 48 h after hypoxia–ischemia and maintained hypothermic (core temperature 33.5 °C) between 2–26 h. Physiological parameters were compared between groups with Mann–Whitney at each time point.

### 2.6 Argon delivery

**Fig. 2:**
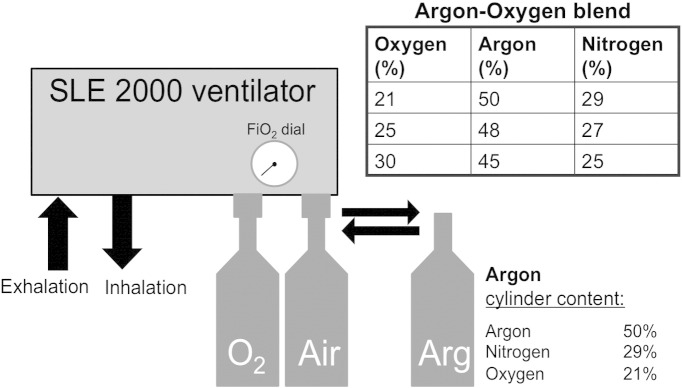
Argon delivery. Piglets were ventilated via an SLE2000 infant ventilator (SLE Ltd., Surry, UK) which has both oxygen and air supply inlets with an oxygen blender. Each cylinder contained 4800 l of compressed gas comprising 50% argon, 21% oxygen and 29% nitrogen. During Argon treatment, the air supply was switched with an Argon cylinder connected inline with the ventilator. When FiO_2_ was increased to maintain piglet peripheral oxygen saturations and PaO_2_ within normal parameters, the ventilator blender increased the oxygen delivery and decreased the delivery from the Argon cylinder, resulting in a reduction in inspired Argon and Nitrogen concentrations as shown. No piglet required more than 30% FiO_2_ therefore all piglets in the Argon + Cooling group received a minimum of 45% Argon throughout treatment.

In those randomized to Cooling + Argon, 45–50% Argon was delivered through the ventilator from 2–26 h (Fig. 1). Argon Gas was obtained from Air Liquide Ltd. (Manchester UK). Piglets were ventilated via an SLE 2000 infant ventilator (SLE Ltd., Surrey, UK), which has both oxygen and air supply inlets with an oxygen blender. During Argon treatment, the air supply was switched with an Argon cylinder connected inline with the ventilator (Fig. 2). When FiO_2_ was increased to maintain piglet peripheral oxygen saturations and PaO_2_ within normal parameters, the ventilator blender increased the oxygen delivery and decreased delivery from the Argon cylinder, resulting in a small reduction in inspired Argon and Nitrogen concentrations. No piglet required more than 30% FiO_2_ therefore all piglets in the Cooling + Argon group received a minimum of 45% Argon throughout treatment.

### 2.7 aEEG/EEG acquisition

After surgical preparation, multichannel six-lead EEG monitoring (Nicolet, Care Fusion, Wisconsin, USA) was acquired at baseline and throughout the periods between MRS data acquisitions. Filtered amplitude-integrated EEG recordings were classified according to the pattern classification (Hellström-Westas et al., 1995). A score of 0 was flat trace; 1, continuous low voltage; 2, burst suppression; 3, discontinuous normal voltage; and 4, continuous normal voltage, at baseline and then every hour after hypoxia–ischemia.

### 2.8 Brain histology

**Fig. 3:**
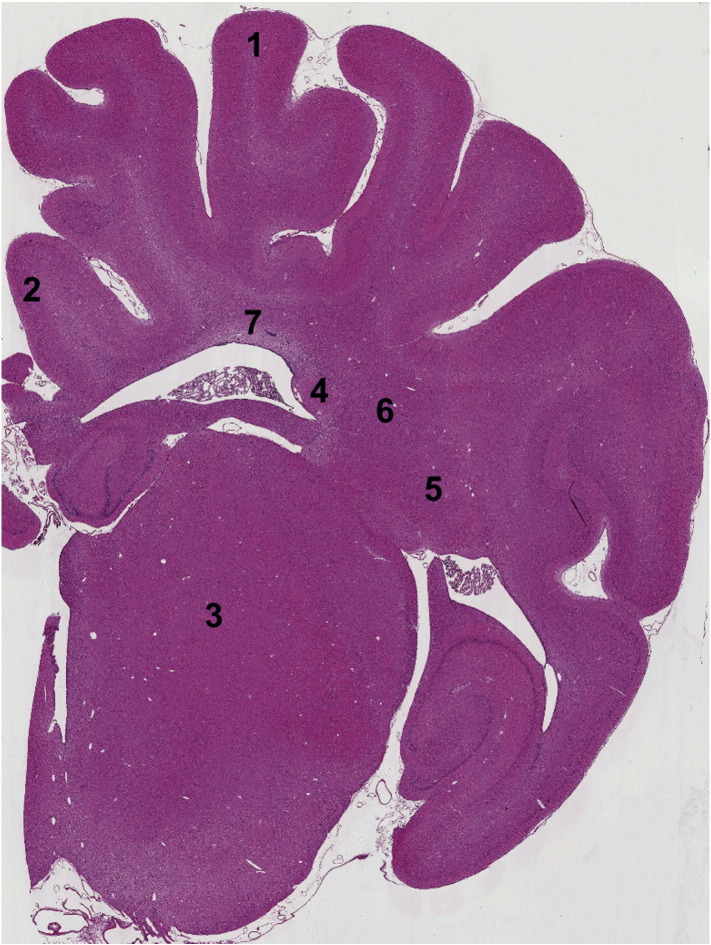
Histology. Representative piglet brain photomicrograph indicating brain regions assessed for TUNEL immunohistochemistry. 1) Sensorimotor Cortex, sTEX; 2) Cingulate Cortex, cTEX; 3) Thalamus, THAL; 4) Caudate Nucleus, CDT; 5) Putamen, PTMN; 6) Internal Capsule, IC; 7) Periventricular white matter, pvWM.

At 48 h after hypoxia–ischemia, piglets were euthanized with pentobarbital, the brain was fixed by cardiac perfusion with cold 4% paraformaldehyde, dissected out and post-fixed at 4 °C in 2% paraformaldehyde for 7 days. Coronal slices (5 mm thick) of the right hemisphere, starting from anterior to the optic chiasma, were embedded in paraffin, sectioned to 8-μm thickness and stained with hematoxylin and eosin to validate the bregma for analysis. For each animal, 2 sections (bregma 00 and − 2.0) were stained and 7 different regions in the brain were examined (Fig. 3).

To assess cell death, brain sections were stained for nuclear DNA fragmentation using histochemistry with transferase mediated biotinylated d-UTP nick end-labeling (TUNEL) as previously described (Robertson et al., 2013). Briefly, TUNEL sections were pretreated in 3% hydrogen peroxide, subjected to a protease K pre-digestion (Promega, Southampton, UK) and incubated with TUNEL solution (Roche, Burgess Hill, UK). TUNEL was visualized using avidin-biotinylated horseradish complex (ABC, Vector Laboratories, Peterborough, UK) and diaminobenzidine/H_2_O_2_ (DAB, Sigma, Poole, UK) enhanced with CoSO_4_ and NiCl_2_. TUNEL sections were dehydrated and cover-slipped with DPX (VWR, Leighton Buzzard, UK). For each animal and brain region, TUNEL-positive nuclei were counted at two levels and from 7 regions (Fig. 3) by an investigator blind to the treatment group and the average converted into counts per mm^2^.

### 2.9 Statistical methods

#### 2.9.1 MRS

All analyses were performed using the SAS JMP® v11.0.0 software. A statistical model was fitted to the ratios NTP/epp, PCr/Pi and Lac/NAA. An analysis of variance (ANOVA) model was fitted and the differences in the means on the log scale for the two treatment groups (Cooling versus Cooling + Argon) were estimated from the model at each of the three time points with 95% confidence intervals (C.I.s) for the differences. The differences in treatment group means are shown graphically using 95% Least Significant Difference error bars. Corrections for multiple measurements were not made.

#### 2.9.2 EEG

Following the baseline scoring, scores were obtained hourly until 48 h from hypoxia-ischemia. Each subject's scores were averaged over the following periods; 0–6 h, 7–12 h, 13–18 h, 19–24 h, 25–30 h, 31–36 h, 37–42 h and an analysis of variance model fitted to the mean scores. The differences in the means between the two treatment groups (Cooling plus Argon versus Cooling) were estimated from the model at each of the seven timepoints with 95% confidence intervals (C.I.s) for the differences.

#### 2.9.3 TUNEL

An analysis of variance model was fitted to the mean counts to give an estimate of the expected counts per mm^2^. The overall difference between the means for the two treatment groups, and treatment differences across regions are presented with 95% C.I.s and graphically using 95% Least Significant Difference error bars.

## 3 Results

There were 10 animals in the Cooling group and 8 animals int he Cooling + Argon group. One piglet (Cooling + Argon) was lost prior to 48 h due to cardiac arrest. Another piglet (Cooling + Argon) was excluded because the cooling mattress malfunctioned and the piglet was normothermic between 7–9 h; in addition a fault was noted in the ventilator and Argon delivery was not assured for a period of several hours.

### 3.1 Physiological data and insult severity

There were no significant intergroup differences between groups in bodyweight, postnatal age and baseline physiological measures apart from the base excess, which was more alkaline in the Cooling + Argon group at baseline (Table 1). There was no difference in the hypoxic–ischemic insult severity between the two groups (Table 1). There was no difference between groups for volume replacement and inotrope use following hypoxia-ischemia (Table 2).

### 3.2 Argon usage

Fifty-four Argon cylinders were used for this study. Each cylinder contained 4800 l of compressed gas comprising 50% argon; 21% oxygen and 29% nitrogen. Each cylinder lasted 5.5 h — around 872 l/h or 14.5 l/min was delivered to the SLE ventilator. The SLE ventilator, however, vents 8–9 l of air/min leaving the gas delivery to the pig of 4–5 l/min.

### 3.3 MRS analysis showed improved PCr/Pi, NTP/epp at 48 h and lower white matter Lac/NAA at 24 and 48 h

**Fig. 4:**
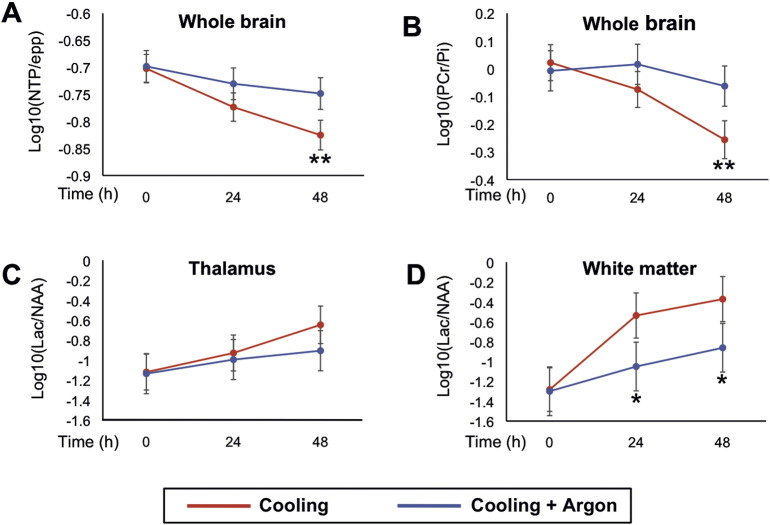
Magnetic resonance spectroscopy of the brain at baseline, 24 and 48 h after hypoxia-ischemia. Least squares means plot with 95% Least Significant Difference (LSD) bars for the NTP/epp and PCr/Pi in whole-forebrain, and Lac/NAA in thalamus and white matter; non-overlapping bars show evidence of a significant difference. Whole-forebrain NTP/epp (A) and PCr/Pi (B) means were significantly higher in the Cooling + Argon group compared to Cooling at 48 h post-HI. White matter Lac/NAA was significantly lower in the Cooling + Argon group compared to Cooling at both 24 and 48 h (p = 0.03 and p = 0.04 respectively). Thalamic ^1^H MRS showed no difference between groups at any time point. *p < 0.05, **p = 0.01. epp = exchangeable phosphate pool; Lac = lactate; NAA = N-acetyl aspartate; Thal = thalamic; WM = white matter; HI = hypoxia–ischemia.

The least squares means plots and 95% Least Significant Difference (LSD) bars for the NTP/epp, PCr/Pi and Lac/NAA (on log10 scale) in thalamus and white matter are shown in Fig. 4. The differences in the means and C.I.s are shown in Table 3. NTP/epp and PCr/Pi means were significantly higher at 48 h in Cooling + Argon compared to the Cooling group (p = 0.01 for both). White matter Lactate/NAA was significantly lower in the Cooling + Argon compared to the cooling group at 24 and 48 h (p = 0.03 and p = 0.04 respectively). Thalamic ^1^H MRS showed no differences between groups at any time point.

### 3.4 aEEG recovery was faster in the Cooling + Argon group

**Fig. 5:**
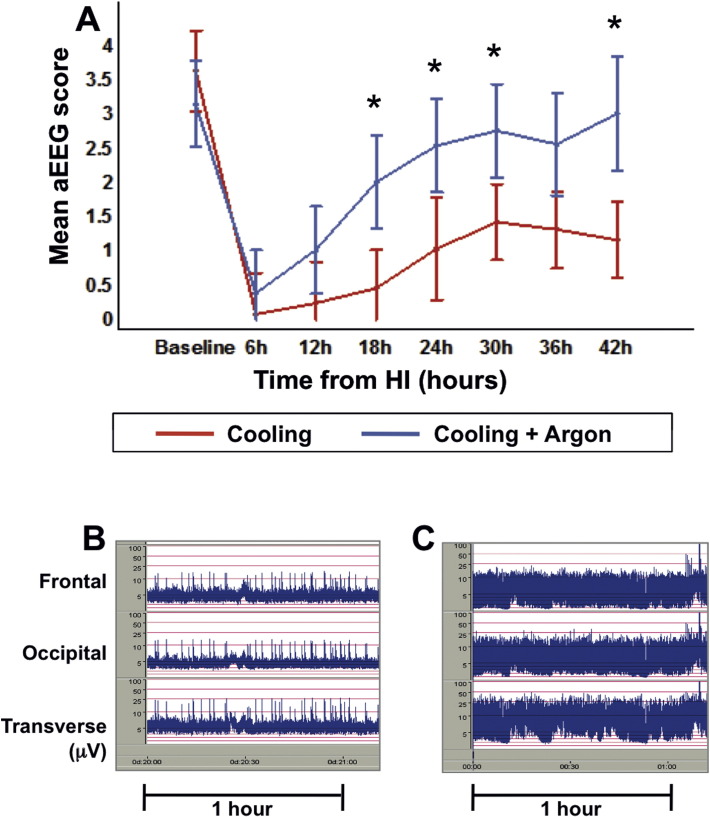
Amplitude-integrated electroencephalogram (aEEG). The group mean hourly aEEG scores were significantly higher in the Cooling + Argon group versus Cooling alone, from 18 h post-HI onwards, indicating faster recovery of brain electrical activity towards normal. Panel A shows grouped mean hourly aEEG scores per 6-hour period with 95% Least Significant Difference (LSD) where non-overlapping bars show evidence of a significant difference. Representative aEEG traces are shown at 24 h post-HI for the Cooling (B) and Cooling + Argon (C) groups. aEEG = amplitude-integrated EEG; HI = hypoxia–ischemia. *p < 0.05.

The group mean hourly aEEG scores were significantly higher (p < 0.05) in the Cooling + Argon versus Cooling group from 18 h post hypoxia–ischemia onward, indicating faster recovery of brain electrical activity towards normal with Argon-augmented cooling. The overall difference between the means for the two treatment groups, and treatment differences for each time interval are presented with 95% C.I.s (Table 4) and graphically using 95% Least Significant Difference error bars (Fig. 5).

### 3.5 Cooling + Argon decreased TUNEL positive cell death at 48 h

**Fig. 6:**
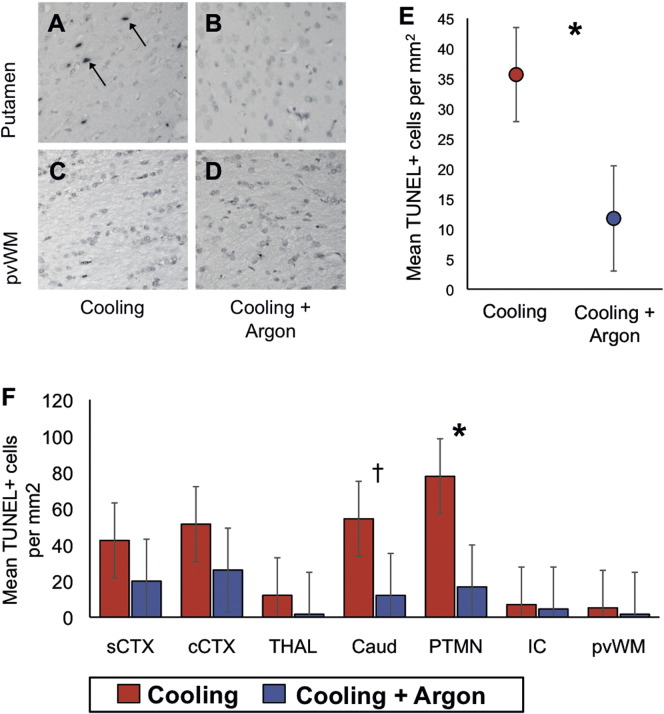
TUNEL histology. Co-treatment with 45-50% Argon decreased TUNEL-positive cell death at 48 h after a hypoxic ischemic insult when compared to cooling alone. Representative sections are shown at × 20 magnification from the same animal in the Cooling (*left* column, A and C) and Cooling + Argon (*right* column, B and D) from the putamen (PTMN) and periventricular white matter (pvWM). There was an overall decrease in the estimated mean TUNEL-positive cells per mm^2^ (pooled across region and R0/R1 levels) in the Cooling + Argon group versus Cooling alone (E). On regional assessment, there was a significant decrease in TUNEL-positive cells in the Putamen and evidence of a trend in the Caudate in the Cooling + Argon group versus Cooling alone (F). *p < 0.01, †p = 0.07. Sensorimotor cortex = sTEX; Cingulate cortex = cTEX; Thalamus = THAL; Caudate nucleus = CDT; Putamen = PTMN; Internal capsule = IC; Periventricular white matter = PvWM.

Representative photomicrographs of TUNEL staining in the putamen and periventricular white matter are shown for Cooling and Cooling + Argon are shown in Fig. 6A–D. The 95% C.I.s are shown in Table 5. There was evidence of an overall difference between the means of the Cooling versus Cooling + Argon treatment groups (p = 0.018) with the estimated cells per mm^2^ 20.9 points lower (95% C.I. 3.7–38.2) for the Cooling + Argon versus Cooling alone. The estimated mean cells per mm^2^ for the two treatment groups by region are shown in Fig. 6E. The region showing the largest difference in cell death was the putamen with a mean difference of 61.0 cells per mm^2^ (p = 0.01). The caudate showed a mean difference of 42.2 cells per mm^2^ (p = 0.07) (Fig. 6F).

## 4 Discussion

Compared to cooling alone, we observed improved cerebral protection with a combination of cooling and 45–50% Argon started at 2 h after hypoxia–ischemia and continued for 24 h in our newborn piglet model of perinatal asphyxia. The addition of Argon to cooling increased whole brain ATP and PCr at 48 h (^31^P MRS NTP/epp and PCr/Pi) and reduced the secondary rise in white but not gray matter Lac/NAA on localized ^1^H MRS at 24 and 48 h. Compared to cooling, the combined therapy led to a faster aEEG recovery from 18 h after hypoxia–ischemia and a reduction in cell death on TUNEL staining over seven brain regions combined and in the putamen specifically.

Our MRS biomarkers correlate with injury severity after hypoxia–ischemia in the piglet (Lorek et al., 1994, Penrice et al., 1997) and outcome in infants with NE (Robertson et al., 1999). Higher ATP on ^31^P MRS in infants with NE is associated with better long-term outcome in clinical studies (Azzopardi et al., 1989); we saw higher levels of ATP with Argon-augmented cooling compared to cooling alone. High levels of thalamic lactate/NAA on MRS in neonates in the first month after birth are predictive of a poor 12–18 month neurodevelopmental outcome (Robertson et al., 1999, Thayyil et al., 2010); we saw reduced Lac/NAA on white matter MRS with Argon-augmented cooling compared to cooling alone. The aEEG background voltage and rate of aEEG recovery after HI are also predictive of neurodevelopmental outcome in NE (van Rooij et al., 2005) even in babies undergoing therapeutic hypothermia, with a positive predictive value of an abnormal background pattern of 0.82 at 48 h (Csekő et al., 2013). In our study, electrical activity on the aEEG normalized more rapidly with Argon and cooling from 18 h onwards compared to cooling alone.

Argon was not associated with any cardiovascular or blood pressure changes during the 24 h delivery period and was well tolerated by all piglets. This is in keeping with the physiological stability seen in a recent piglet safety study of inhaled Argon in concentrations up to 80% (Alderliesten et al., 2014). In our study, Argon was straightforward to deliver through a standard neonatal SLE 2000 ventilator with no requirement for a scavenging system, unlike Xenon (Faulkner et al., 2012). Despite its 200 fold higher cost and scarce supply, Xenon has been more widely studied than Argon and has shown significant brain protection in adult (Schmidt et al., 2005) and neonatal (Ma et al., 2005) pre-clinical studies of hypoxia–ischemia, particularly when combined with cooling (Chakkarapani et al., 2010, Faulkner et al., 2011). Clinical trials of Xenon as an additional treatment to hypothermia for NE are underway in the UK (TOBYXe; NCT00934700 and Cool Xenon study; NCT01545271). Although Xenon is an anesthetic at atmospheric pressure and Argon is not, both noble gases share the important attribute of good blood brain barrier penetration and fast onset, which are vital properties for neuroprotection.

Protective effects of Argon have been seen in *in vitro* excitotoxic and hypoxic models (Coburn et al., 2008, Jawad et al., 2009, Loetscher et al., 2009). An *in vivo* adult rodent model of transient middle cerebral artery occlusion (MCAO) exposed to 50% Argon 1 h after hypoxia–ischemia showed a significant overall reduction in infarct volume compared to no Argon; this protection was most marked in the cortex and basal ganglia (Ryang et al., 2011). Another MCAO study of found similar protection in the cortex with 50% Argon, but increased subcortical damage and no improvement in neurological deficit (David et al., 2012). In our Argon piglet study we observed higher levels of ATP across the whole brain on ^31^P MRS but more protection in the white matter than gray matter voxel on ^1^H MRS. This is supported by the finding of improved electrographic recovery on aEEG, as the white matter voxel captures the dorsal aspect of the brain corresponding to EEG lead placement. MRS voxel position may explain why gray matter protection was not seen; the gray matter MRS voxels were centered on both thalami and so did not sample metabolism in the putamen where strong histological cell protection was seen. As well as significant histological protection in the putamen, there was a trend towards protection in the caudate but not in the thalamus. Histological protection was not seen in the white matter regions — the internal capsule and periventricular white mater (see Table 5 and Fig. 5); this may relate to the lower levels of cell death with cooling and less opportunity for white matter protection with the addition of Argon. Combining all brain regions there was significant protection with the addition of Argon to cooling. A partial volume effect of the white matter MRS voxel may have resulted in MRS sampling of adjacent brain regions including areas of gray matter. Protection observed with Argon in our current study appears more robust than with Xenon in the same model in 2011 with a similar insult (Faulkner et al., 2011); in this previous study we observed no statistically significant difference between Xenon-augmented cooling and cooling alone, although differences were seen when combined therapy was compared with no therapy. Interestingly, histological protection was also observed with Xenon augmented cooling in the putamen (as seen with Argon) and the cortex, however this protection was only significant when compared to control animals not receiving cooling (Faulkner et al., 2011).

There are several possible mechanisms thought to mediate Argon's brain protection. As Argon (atomic number 18) is smaller than Xenon (atomic number 54), this may change its binding sites. Argon triggers gamma-aminobutyric acid (GABA) neurotransmission by acting at the benzodiazepine binding site of the GABA_A_ receptor (Abraini et al., 2003), however this is seen typically under hyperbaric conditions and the role of this pathway is unclear in the immature brain in which GABA receptor activation is excitatory (Ben-Ari et al., 2012). Nevertheless, the activation of GABA receptors in mature brain has been shown to be protective (Schwartz-Bloom and Sah, 2001). Argon has also been seen to have oxygen-like properties where it increased survival in animals under hypoxic conditions (Soldatov et al., 2008); this may confer mitochondrial protection in the post ischemic period. Anti-apoptotic signaling is an important mechanism of Argon's brain protection. Like Xenon, Argon increases expression of cell survival proteins; those specific to Argon are increased expression of Bcl-2 (Zhuang et al., 2012) and enhancement of the ERK 1/2 activity in astrocytes, neurons and microglia by direct activation of the MEK/ERK 1/2 pathway (Fahlenkamp et al., 2012). Argon reduces heat shock protein expression (Ulbrich et al., 2015). Argon does not appear to influence NMDA receptors or potassium channels (Brücken et al., 2014), which are two important mechanisms of Xenon and hypothermic protection. The additive protection seen with the combination of Argon and cooling suggests that Argon targets complementary cell protection cascades to cooling, unlike Xenon, which targets more similar cascades to cooling. For example, one important action of cooling is to reduce glycine release after hypoxia–ischemia (Illievich et al., 1994). Unlike Xenon, Argon has been shown to have no effect on NMDA receptors at high or low glycine levels (Harris et al., 2013). We did not study the effect of Argon-augmented cooling on neuroinflammation. A recent rodent study found elevated expressions of several inflammatory cytokines (IL-1β, IL-6, iNOS) and an increased expression of growth factors (TGFβ, NGF, VEGF) following transient MCAO + Argon compared to MCAO + placebo (Fahlenkamp et al., 2014).

The study has some limitations. We observed that the Cooling + Argon group's blood pH was more alkaline at baseline than the Cooling group. It is unclear why this occurred, however, both levels are within the normal range for piglets and all included piglets appeared healthy, with normal baseline blood lactate levels. Some *in vitro* studies suggest there may be an additional effect of Argon at higher concentrations than 50% (Ulbrich et al., 2014); we chose 50% as it has shown maximal benefit in some studies (Loetscher et al., 2009) and allows for the inspired oxygen to be increased if required to maintain oxygen saturations. In both Cooling and Argon + Cooling groups we saw higher blood glucose during cooling; this may be due to the cooling itself as cooling has been associated with increased blood glucose variability and greater insulin requirements compared to the post-rewarming normothermic phase (Escolar et al., 1990). Finally, no formal corrections for multiple measurements of MRS were made; the p values are indicators of a signal that is likely to have biological and outcome significance.

In summary, this is the first study to show augmentation of hypothermic neuroprotection in a neonatal pre-clinical large animal model of birth asphyxia. 45–50% Argon was practical and easy to deliver and was not associated with any physiological differences during the 24 h exposure with cooling. Protection was assessed using biomarkers (MRS and aEEG), which are strongly predictive of outcome in babies with NE. The overall brain cell death was reduced with the addition of 24 h Argon to cooling and this protection was evident with a two-hour delay starting therapy. Argon may be an affordable and practical therapy to augment hypothermic brain protection in babies with NE.

## Figures and Tables

**Table 1:** Baseline group data and physiological variables throughout the studies.

Parameter	Cooling Mean (SD)	Cooling + Argon Mean (SD)	p value
Postnatal age (h)	29.1 (7.7)	31.0 (8.9)	0.51
Body weight (g)	1978 (109)	1925 (89)	0.19
Duration of HI (min)	24.2 (2.8)	23.0 (1.9)	0.30
Insult severity (10^− 2^) measured from acute energy depletion (AED)	9.47 (3.14)	8.63 (2.62)	0.63

Heart rate (min^− 1^)			
Baseline	175 (20)	165 (23)	0.36
End of insult (time 0)	184 (26)	178 (25)	0.92
1–2 h after time 0	178 (19)	163 (24)	0.12
2–26 h after time 0	154 (14)	158 (20)	0.50
26–48 h after time 0	166 (29)	178 (21)	0.54

Mean arterial blood pressure (mm Hg)			
Baseline	47 (8)	45 (7)	0.47
End of insult (time 0)	48 (16)	54 (19)	0.53
1–2 h after time 0	47 (10)	46 (9)	0.70
2–26 h after time 0	46 (3)	46 (7)	0.81
26–48 h after time 0	54 (7)	53 (9)	0.96

Rectal temperature (°C)			
Baseline	38.2 (0.7)	38.2 (0.6)	0.96
End of insult (time 0)	37.8 (0.6)	37.9 (0.6)	0.33
1–2 h after time 0	38.2 (0.6)	38.2 (0.7)	0.81
2–26 h after time 0	33.7 (0.2)	34.2 (1.4)	0.85
26–48 h after time 0	37.0 (0.8)	37.2 (0.3)	0.85

PaO2 (kPa)			
Baseline	15.3 (6.2)	22.4 (20.0)	0.92
Insult nadir	4.9 (1.1)	6.3 (4.5)	0.77
12 h after time 0	15.2 (4.8)	12.1 (3.7)	0.36
24 h after time 0	20.2 (5.6)	15.1 (3.8)	0.13
48 h after time 0	23.5 (14.9)	23.3 (13.9)	0.63

PaCO_2_ (kPa)			
Baseline	5.2 (1.4)	5.0 (0.6)	0.67
Insult nadir	5.3 (1.1)	4.6 (1.1)	0.10
12 h after time 0	5.7 (1.5)	6.9 (2.5)	0.50
24 h after time 0	5.5 (3.1)	4.5 (2.0)	0.96
48 h after time 0	6.0 (1.8)	5.7 (2.0)	0.63

Blood pH			
Baseline	7.43 (0.06)	7.26 (0.68)	0.36
Insult nadir	7.35 (0.10)	7.39 (0.08)	0.12
12 h after time 0	7,42 (0.10)	7.38 (0.14)	0.63
24 h after time 0	7.44 (0.19)	7.52 (0.20)	0.29
48 h after time 0	7.35 (0.09)	7.42 (0.14)	0.27

Base excess (mmol/l)			
Baseline	1.2 (4.8)	7.8 (4.7)	**0.02**
Insult nadir	− 4.1 (3.5)	− 2.0 (4.0)	0.33
12 h after time 0	2.2 (3.5)	4.1 (3.0)	0.31
24 h after time 0	0.6 (5.1)	2.1 (3.9)	0.87
48 h after time 0	− 1.1 (4.1)	1.9 (4.2)	0.34

Lactate (mmol/l)			
Baseline	4.28 (2.32)	2.62 (1.23)	0.08
Insult nadir	8.85 (2.38)	7.22 (2.17)	0.21
12 h after time 0	2.83 (1.46)	3.11 (2.21)	0.77
24 h after time 0	3.80 (3.53)	3.74 (1.92)	0.25
48 h after time 0	2.27 (2.30)	1.23 (0.63)	0.24

Glucose (mmol/l)			
Baseline	6.6 (1.7)	5.6 (1.8)	0.16
Insult nadir	9.1 (2.3)	8.9 (2.0)	0.80
12 h after time 0	12.8 (4.7)	9.2 (4.8)	0.08
24 h after time 0	16.3 (7.8)	13.2 (7.4)	0.56
48 h after time 0	8.5 (3.9)	7.2 (3.8)	0.48

Time zero was set at the time of reperfusion/resuscitation. Mean and standard deviation (SD) values are presented for the two groups; (i) Cooling (n = 10) and (ii) Cooling + Argon (n = 8). Analysis using Mann–Whitney test indicated that there was no evidence of a difference between the two groups for any of the outcomes at any of the time-points apart from the baseline base excess was more alkaline in the Cooling + Argon group. Insult severity was estimated by calculating the time integral of the change in NTP/epp during HI and the first 60 min of resuscitation.

**Table 2:** Volume and inotrope requirements.

Treatment	Cooling	Cooling + Argon	p value
Median (IQR)	
Saline bolus ml/kg	0 (0,0)	1 (0,1)	NS
Dopamine (μg/kg/min)	12.8 (8.9, 16.0)	17.3 (10.5, 18.5)	0.10
Dobutamine (μg/kg/min)	0 (0.1)	8 (0,11)	0.14
Adrenaline (ng/kg/min)	0 (0.7)	0 (0,6)	NS

Mean (IQR) values for total volume replacement and dopamine, dobutamine and adrenaline infusions. Analysis using Kruskal–Wallis equality of populations rank test revealed no evidence of a statistically significant difference between groups for volume replacement and inotrope use. NS = not significant.

**Table 3:** Summary of differences between Cooling and Cooling + Argon MRS results at 48 h.

	Difference in means (log 10 scale) Cooling–Cooling + Argon	95% C.I. for difference (log scale)	Ratio of means (original scale)	95% C.I. for ratio (original scale)	p-Value
NTP/epp	− 0.077	− 0.13 to − 0.02	0.84	0.74 to 0.95	**p** **<** **0.01**
PCr/Pi	− 0.194	− 0.33 to − 0.05	0.64	0.46 to 0.89	**p** **<** **0.01**
Thalamus Lac/NAA	0.259	− 0.13 to 0.65	1.82	0.74 to 4.45	0.19
White matter Lac/NAA	0.490	0.02 to 0.96	3.09	1.04 to 9.18	**0.04**

The Cooling + Argon group showed higher levels of NTP/epp and PCr/Pi and lower white matter Lac/NAA compared to Cooling alone. epp = exchangeable phosphate pool; PCr = phosphocreatine; Pi = inorganic phosphate; Lac = lactate; NAA = N acetyl aspartate. Bold emphasis indicates statistical significance.

**Table 4:** Differences between the Cooling + Argon and Cooling group aEEG scores at the different time points.

Time interval relative to HI	Difference in mean aEEG (Cooling + Argon–Cooling)	Standard error of the difference	Lower 95% C.I. for difference	Upper 95% C.I. for difference	p value
Baseline (before HI)	− 0.482	0.614	− 1.701	0.736	0.434
0–6 h	0.299	0.614	− 0.920	1.517	0.628
7–12 h	0.773	0.614	− 0.446	1.991	0.211
13–18 h	1.542	0.625	0.301	2.782	**0.015**
19–24 h	1.502	0.718	0.076	2.927	**0.039**
25–30 h	1.324	0.625	0.084	2.565	**0.037**
31–36 h	1.244	0.662	− 0.069	2.558	0.063
37–42 h	1.833	0.713	0.419	3.248	**0.012**

The Argon + Cooling group showed evidence of faster recovery of electrical activity compared to Cooling from the time period 13–18 h after hypoxia–ischemia onwards (p < 0.04 for all intervals except for p = 0.06 for 31–36 h). HI: hypoxia–ischemia. Bold emphasis indicates statistical significance.

**Table 5:** Differences between the Cooling + Argon and Cooling group TUNEL counts for all seven brain regions and overall.

Area	Difference in mean TUNEL count (Cooling–Cooling + Argon)	Standard error of the difference	Lower 95% C.I. for difference	Upper 95% C.I. for difference	p value
Sensorimotor cortex	22.4	23.3	− 23.5	68.4	0.338
Cingulate cortex	25.3	23.3	− 20.7	71.3	0.279
Thalamus	10.4	23.3	− 35.5	56.4	0.655
Caudate	42.2	23.3	− 3.8	88.2	0.072
Putamen	**61.0**	**23.3**	**15.0**	**107.0**	**0.010**
Internal capsule	2.4	23.3	− 43.5	48.4	0.917
Periventricular white matter	3.5	23.3	− 42.5	49.5	0.880
Overall	**20.9**	**8.8**	**3.7**	**38.2**	**0.018**

The overall cell death in 7 regions was reduced in the Argon + Cooling group compared to the Cooling group, with the largest difference seen in the putamen and in the caudate. Bold emphasis indicates statistical significance.
